# MR Imaging Analysis of Non-Measurable Enhancing Lesions Newly Appearing after Concomitant Chemoradiotherapy in Glioblastoma Patients for Prognosis Prediction

**DOI:** 10.1371/journal.pone.0166096

**Published:** 2016-11-11

**Authors:** Bo Ram Kim, Seung Hong Choi, Tae Jin Yun, Soon-Tae Lee, Chul-Kee Park, Tae Min Kim, Ji-Hoon Kim, Sun-Won Park, Chul-Ho Sohn, Sung-Hye Park, Il Han Kim

**Affiliations:** 1 Department of Radiology, Seoul National University Hospital, Seoul, Republic of Korea; 2 Department of Neurology, Seoul National University Hospital, Seoul, Republic of Korea; 3 Department of Neurosurgery, Seoul National University Hospital, Seoul, Republic of Korea; 4 Department of Internal Medicine, Seoul National University Hospital, Seoul, Republic of Korea; 5 Department of Radiology, Boramae Medical Center, Seoul, Republic of Korea; 6 Department of Pathology, Seoul National University Hospital, Seoul, Republic of Korea; 7 Department of Radiation Oncology, Seoul National University Hospital, Seoul, Republic of Korea; George Washington University, UNITED STATES

## Abstract

**Purpose:**

To analyze the enhancement patterns and apparent diffusion coefficient (ADC) values of non-measurable surgical cavity wall enhancement pattern, newly appearing after completion of standard concurrent chemoradiotherapy (CCRT) with temozolomide in glioblastoma patients for the prognosis prediction.

**Materials and Methods:**

From January 2010 to April 2014, among 190 patients with histopathologically confirmed glioblastoma, a total of 33 patients with non-measurable wall enhancement on post-CCRT MR imaging were enrolled and divided into two subgroups: non-progression (n = 18) and progression groups (n = 15). We analyzed the wall enhancement patterns, which were categorized into three patterns: thin, thick and nodular enhancement. ADC values were measured in the enhancing portions of the walls. The progression-free survival (PFS) related to the wall enhancement was analyzed by Kaplan-Meier analysis, and survival curves were compared using the log-rank test.

**Results:**

Statistically significant differences in the surgical cavity wall enhancement patterns was shown between the progression and non-progression groups (*P = 0*.*0032*). Thin wall enhancement was more frequently observed in the non-progression group, and thick or nodular wall enhancement were observed in the progression group (*P = 0*.*0016*). There was no statistically significant difference in the mean ADC values between the progression and non-progression groups. The mean PFS was longer in patients with thin wall enhancement than in those with nodular or thick wall enhancement (35.5 months vs. 15.8 months, *P = 0*.*008*).

**Conclusion:**

Pattern analysis of non-measurable surgical cavity wall enhancement on post-CCRT MR imaging might be useful tool for predicting prognosis of GBM patient before clear progression of non-measurable disease.

## Introduction

Glioblastoma multiforme (GBM) is characterized by aggressiveness and is represented by proliferation and invasion into brain tissue. This aggressive feature of GBM makes new approaches to the treatment of the ‘cells left behind’ after resection important.[1] The current standard treatment for newly diagnosed GBM is based on concurrent chemoradiotherapy (CCRT) with temozolomide (TMZ) and six cycles of adjuvant TMZ after surgical resection to the extent feasible [2]. Despite the improvements of GBM treatment options, the overall prognosis of GBM remains poor [2–4].

The radiologic response assessment becomes important as treatment options developed. Macdonald et al. [5] published criteria for response assessment in high-grade glioma in 1990. Since then the objective radiologic assessment of tumor response has been based on the contrast-enhancing tumor area that is used as reliable surrogate marker for disease progression. Furthermore, the Response Assessment in Neuro-Oncology (RANO) Working Group revised the response criteria for high-grade gliomas from the perception that the Macdonald criteria had a number of limitations. Despite effort to revise the response criteria, measurement of the tumor around cysts or the surgical cavity is challenging. In RANO response criteria [6], a thin cyst or surgical cavity wall enhancement is considered to indicate a non-measurable lesion, unless any nodular component corresponding to measurable criteria exists.

Recently, clinicians and radiologists have experienced the occurrence of non-measurable enhancing lesions in the surgical cavity wall after CCRT with TMZ in GBM patients who underwent complete resection. Moreover, they observed local progressions at the non-measurable enhancing lesions.

Several studies analyzed recurrence patterns of GBM [7–10], which have shown that most recurrences develop at surgical cavity margin. These studies have focused on the treatment failure pattern that occurred within the original treatment field. Other studies [11–13] evaluated the contrast enhancement pattern on early post-operative MR imaging or MR imaging taken on the period between surgery and adjuvant therapy. Although local progression was commonly observed in the surgical cavity, no clinical studies have evaluated clinical significance of the non-measurable enhancing lesions, newly appearing after completion of the standard CCRT with TMZ.

The purpose of this study was to analyze the enhancement patterns and apparent diffusion coefficient (ADC) values from diffusion-weighed imaging (DWI) of the non-measurable surgical cavity wall enhancements, newly appearing after completion of standard CCRT with TMZ for the prediction of prognosis in GBM patients.

## Materials and Methods

### Ethics Statement

This study was approved by the institutional review board (IRB) of Seoul National University College of Medicine and Hospital. The IRB waived the informed consent requirement from the participants due to the retrospective nature of this study.

### Patient Selection

From January 2010 to April 2014, 190 patients with newly histopathologically confirmed GBM, based on the World Health Organization criteria, who had undergone surgical resection, were selected from the electronic medical database of our institution. The inclusion criteria were as follows: (a) gross total surgical resection; (b) CCRT with TMZ and adjuvant TMZ after complete resection; (c) immediate (up to 48 hours) post-operative contrast enhanced MR imaging demonstrating absence of any post contrast enhancement at the surgical cavity wall; (d) post-CCRT contrast enhanced MR imaging and DWI within three months after the completion of CCRT with TMZ; (e) newly developed non-measurable wall enhancement at the surgical cavity on post-CCRT MR imaging; and (f) serial follow-up MR imaging studies. In total, 46 GBM patients with newly appearing non-measurable surgical cavity wall enhancement were identified, and of those, 13 patients were excluded for the following reasons: (a) definite disease progression, after the completion of CCRT with newly developed or enlarged measurable enhancing lesion, which was defined as a bi-dimensionally measurable, contrast-enhancing lesion with two perpendicular diameters of at least 10 mm [6] on post-CCRT MR imaging (n = 4); (b) seeding lesions developing after the completion of CCRT with TMZ (n = 9)

Finally, a total of 33 patients with newly appearing non-measurable wall enhancement (23 men and 10 women; mean age 54.0 years old [range: 22–72 years]) were enrolled in the present study population and were divided into two groups: non-progression (n = 18) and progression (n = 15) groups, according to disease progression, determined by RANO response assessment criteria within the follow-up period. Ten cases of GBM progression were histopathologically confirmed with surgical resection among the 15 patients in the progression group.

### Image Acquisition

The post-operative MR imaging was acquired within 48 hours to avoid the compounding effect of benign enhancement of the surgical site. The first follow-up MR imaging was performed after the completion of CCRT with TMZ (post-CCRT MR imaging) using one of two MR scanners (Signa HDxt 1.5-T scanner [n = 15], GE Medical Systems, Milwaukee, WI, USA; or Verio 3-T scanner [n = 18], Siemens Medical Solution, Erlangen, Germany). The MR imaging examinations were performed according to a standardized protocol, which included spin-echo or gradient T1 weighted imaging (WI), fast spin-echo T2WI, fluid-attenuated inversion recovery imaging (FLAIR), susceptibility-weighted imaging (SWI), DWI and contrast-enhanced T1WI.

The MR imaging parameters were as follows: 558-650/8-20 ms/70-90°/384 x 192–212 (TR/TE/FA/matrix) for spin-echo T1WI; 27-78/20-47.2 ms/15°/384 x 218–224 for gradientecho T1WI; 4500-5160/91-106.3 ms/90-130°/448-640 x 220 for fast spin-echo T2WI; 9000-9900/97-162.9 ms/90-130°/199-220 x 220 for FLAIR images; and 28/20 ms/15°/448 x 255 for SWI. The other parameters were as follows: section thickness, 1–5 mm with or without a 1-mm gap and field of view (FOV), 178–220 x 178–220 mm. DWI was acquired on the axial plane before contrast injection using a single-shot echo planar imaging technique with 6900-2000/55-95 ms /128-240 x 128-192/220-240 x 220–240 (TR/TE/FOV/matrix), 25–40 sections, 3–4 mm section thickness, a 1-mm intersection gap and a voxel resolution of 1.5 x 1.5 x 3.0–4.0 mm at b value = 0 and 1000 sec/mm^2^. DWI was obtained in three orthogonal directions and was combined into a trace image. Using these data, ADC maps were calculated on a voxel-by-voxel basis with the software incorporated into the MR imaging unit.

Gradient echo T1WI was repeated with same parameters for contrast-enhanced T1WI after injection of a standard dose (0.1 mmol per kilogram of body weight) of gadobutrol (Gadovist, Bayer Schering Pharma, Berlin, Germany) at a rate of 4 mL/sec.

### Qualitative Image Analysis

**Fig 1:**
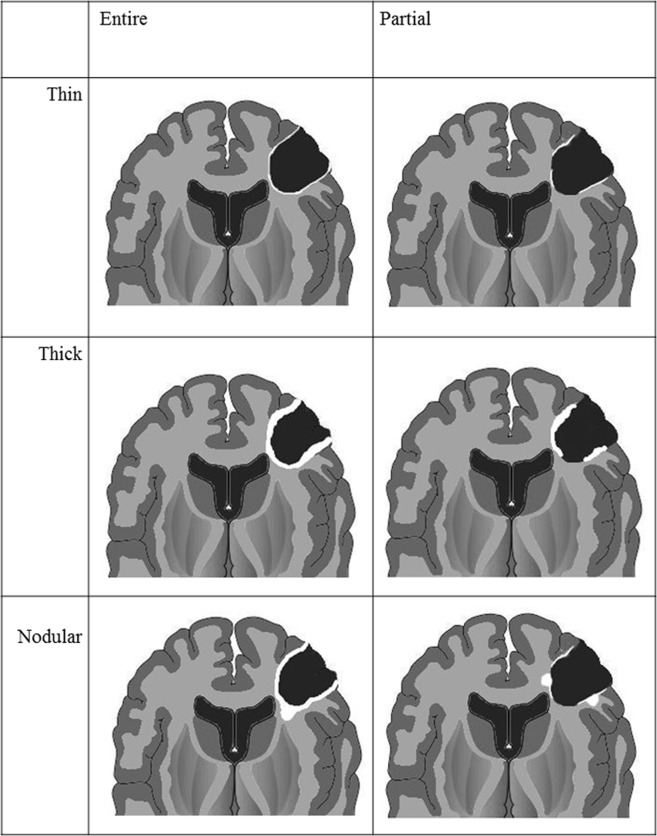
Diagram of surgical cavity wall enhancement patterns.

**Fig 2:**
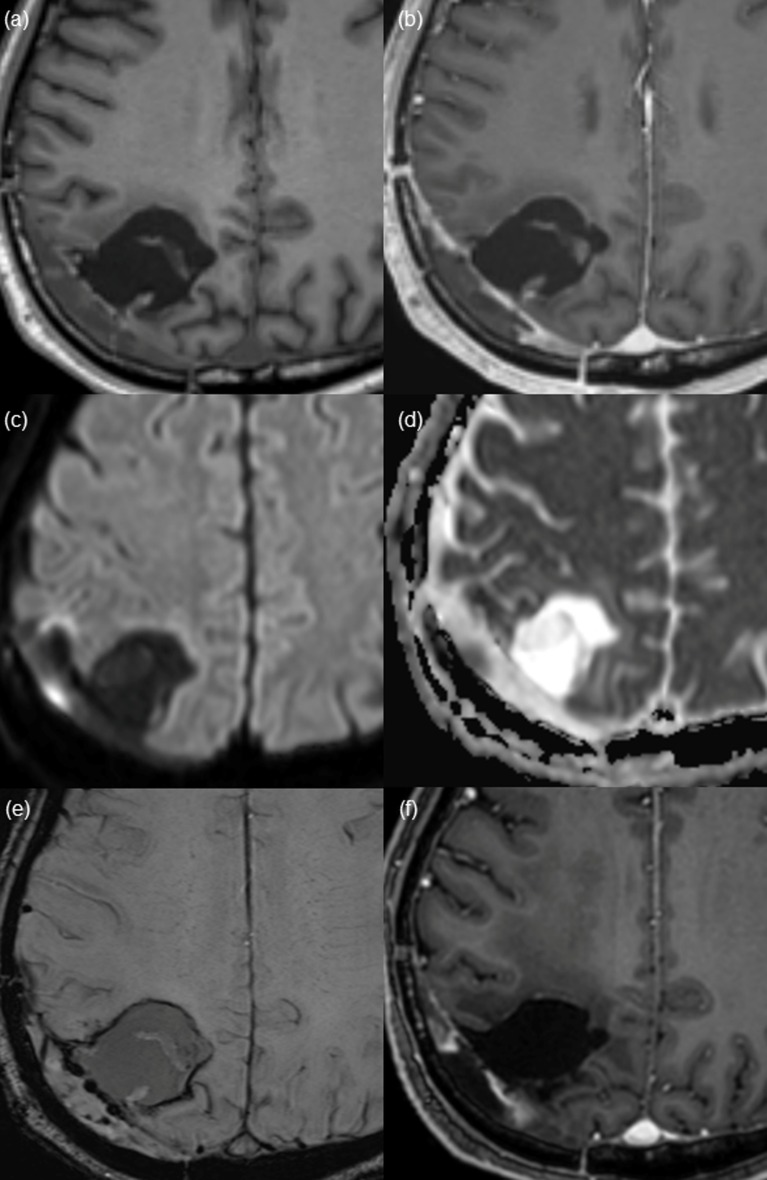
Thin wall enhancement pattern of the surgical cavity found on post-CCRT magnetic resonance (MR) imaging from a 71-year-old patient after gross total resection of glioblastoma. (a and b) Pre- and post-contrast enhanced T1 WI shows thin linear enhancement along the surgical cavity wall. (c) DWI and corresponding (d) ADC map suggest the possibility of diffusion restriction. However, considering the dark signal intensity on SWI (e), high SI on DWI reflects hemorrhage and does not suggest true diffusion restriction. (f) After 20 months, on the last follow up image, no measurable enhanced lesion is shown on contrast-enhanced T1WI.

**Fig 3:**
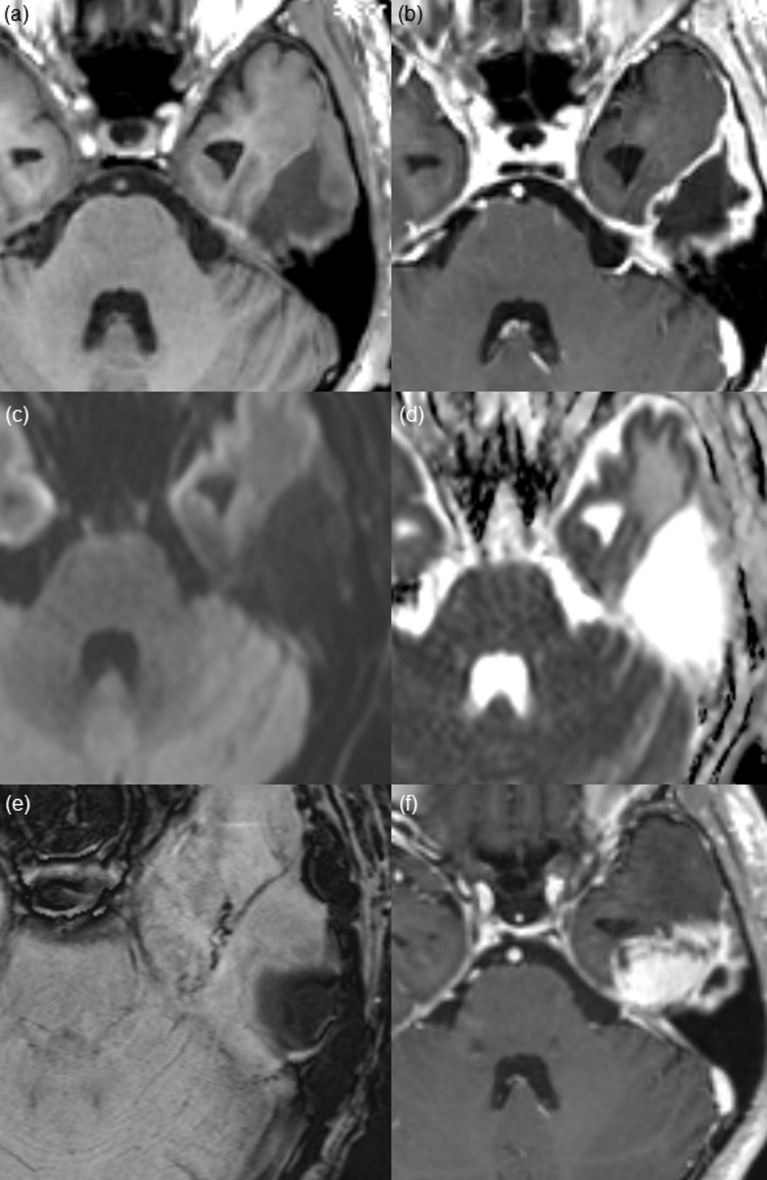
Thick wall enhancement pattern found on post-CCRT MR imaging from a 50-year-old male patient after gross total resection of glioblastoma. (a and b) Pre- and post-contrast enhanced T1 WI shows thick and irregular enhancement along the surgical cavity. (c) DWI and (d) ADC map show no diffusion restriction. Considering the dark signal intensity on (e) SWI, it reflects hemorrhage. (f) On 14-month follow-up contrast-enhanced T1 WI, the surgical cavity has shrunk, and a measurable mass has developed at the inferior aspect of the surgical cavity, which is pathologically confirmed as a recurred glioblastoma.

**Fig 4:**
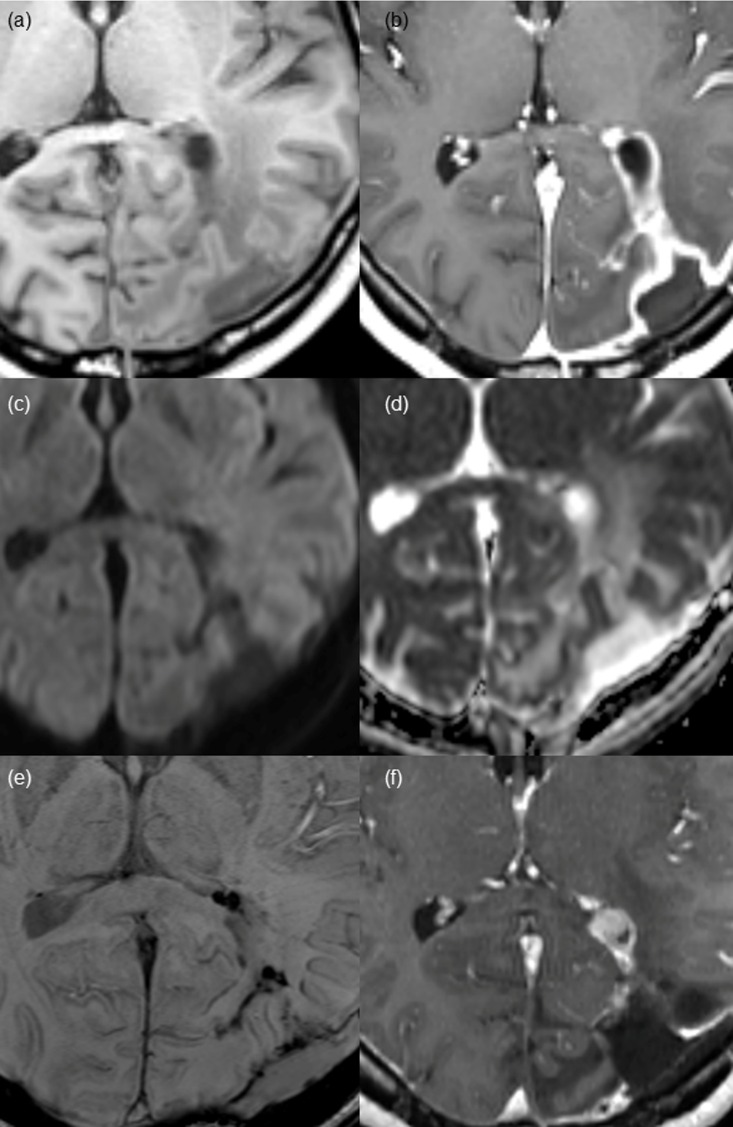
Nodular wall enhancement pattern found on post-CCRT MR imaging in a 36-year-old female patient after gross total resection of glioblastoma. (a and b) Pre- and post-contrast enhanced T1 WI demonstrates a nodular enhancement of 7 mm in the diameter at the anterior margin of the surgical cavity with thick wall enhancement. No demonstrable diffusion restrictions on (c) DWI or (d) ADC map in the enhancing portion. (e) Irregular dark signal intensity is shown along the surgical cavity on SWI, suggestive of hemorrhage. (f) At 15-month follow-up MR imaging, a measurable nodular enhancing lesion appears in the same portion of the surgical cavity, which is pathologically proven recurred glioblastoma.

For each patient, post-CCRT MR imaging was evaluated by two radiologists (S.H.C. and B.R.K.; 13 and 3 years of experience in neuroradiology, respectively), who were blinded to patient prognosis. For qualitative imaging analysis, readers analyzed surgical cavity wall enhancement, which was newly developed on post-CCRT MR imaging. We categorized different non-measurable enhancement patterns in the surgical cavity wall into three groups (Fig 1): thin, thick and nodular wall enhancement. The enhancement pattern of the surgical cavity wall was categorized into thin wall enhancement (Fig 2) when it showed partial or entire wall enhancement of thickness of < 3 mm. Surgical cavity wall enhancement with partial or entire wall enhancement of ≥ 3 mm thickness was considered as thick wall enhancement (Fig 3). Nodular wall enhancement (Fig 4) was assigned when nodular enhancement of ≥ 5 mm in diameter was noted at the surgical cavity, regardless of the presence of thin or thick wall enhancement. Each reader recorded the category of the enhancement patterns, respectively. However, final decision of enhancement pattern category was determined by consensus between two readers in order to resolve the disagreements between two readers and to improve the reproducibility, as Majos et al. [12] suggested.

### Quantitative Image Analysis

ADC values were measured at the enhancing portions in the surgical cavity walls on a picture archiving and communication system workstation. For the measurement, we placed three regions of interests (ROIs) along the enhancing portions and recorded the ADC value from the three ROIs. The average size of ROIs was 6.91± 6.2 mm^2^ (mean ± standard deviation [SD]). We applied normalized ADC (nADC) values to minimize the bias from the use of multiple MR scanners [13, 14], and we calculated average nADC values in the enhancing portion in each patient. The nADC value of each voxel was defined as the ADC value of the voxel divided by the ADC value of the normal periventricular white matter, which was measured at the contralateral side of the surgical cavity. In addition, we evaluated the signal intensity of the surgical cavity margin on SWI as well as phase imaging. The analysis of SWI and phase imaging could determine the presence of hemorrhage in the surgical cavity.

### Patient Follow up and Progression Assessment

The 33 patients were followed up during a median period of 18 months (range, 9–51 months). We assessed the disease progression, using the serial follow up MR imaging and clinical feature. Patients with any one of following criteria were classified as progression group, according to the RANO criteria [6]: (a) significantly increased in size of non-measurable lesion (> 5 mm increase in maximal diameter or ≥ 25% increase in sum of perpendicular diameters of enhancing lesions) and become measurable; (b) developed any new lesion; (c) clear clinical deterioration not attributable to other cause apart from the tumor; (d) failure to return for evaluation as a result of death or deteriorating condition. Additionally, we evaluated the incidence of pseudoprogression, which was diagnosed according to the RANO criteria in all of the patients independently of patient group.

### Statistical Analysis

Statistical analyses were performed with Medcalc software (version 15.6.1 for Microsoft Windows XP/Vista/7/8; MedCalc software, Mariakerke, Belgium). P values less than 0.05 were considered statistically significant and were two sided. Clinical characteristics and categorical data were compared between the progression and non-progression group patients using Fisher’s exact test or the Chi-square test, and Student’s independent t-test was used for non-categorical data. Weighted к-values were used to evaluate interreader agreement of enhancement pattern categorization. The к coefficient in the range of 0.81–1.00 was interpreted as excellent, 0.61–0.80 as substantial, 0.41–0.60 as moderate, 0.21–0.40 as fair, and 0.00–0.20 as poor. In terms of progression-free survival (PFS) related to surgical cavity wall enhancement, survival curves were created using Kaplan-Meier analysis. The difference between survival curves was compared using the results of the log-rank test.

## Results

### Clinical Characteristics of the Patients

The clinical characteristics of 33 patients, including age, Karnofsky performance score, radiation dose, and methylation status of MGMT promoter, are summarized in Table 1. No clinical parameters were statistically significant between the progression and non-progression groups. Local intracranial chemotheraphy using biodegradable casrmustine wafers was not performed in any of the patients.

**Table 1:** Clinical characteristics of patients.

Characteristics	Total	Progression group	Non-progression group	P value
**No. of patients**	33	18	15	
**Age (years, mean ± SD)**	53.9 ± 13.75	55.3 ± 12.36	52.27 ± 15.52	0.371
**Karnofsky performance score**				0.489
**<70**	2 (6%)	2 (11%)	0 (0%)	
**≥70**	31 (94%)	16 (89%)	15 (100%)	
**Radiation dose (Gy)**	59.2 ± 4.97	59.6 ± 4.45	58.7 ± 5.61	0.361
**Methylation status of MGMT promoter**				0.313
**Negative**	13 (39.4%)	9 (50%)	4 (30.8%)	
**Positive**	20 (60.6%)	9 (50%)	11 (73.3%)	
**Pseudoprogression**				0.674
**Negative**	26 (78.8%)	15 (83.3%)	11 (73.3%)	
**Positive**	7 (21.2%)	3 (16.7%)	4 (26.7%)	

In addition, we analyzed the incidence of pseudo-progression after CCRT with TMZ in both the progression and non-progression groups, which showed no statistically significant difference between two groups (*P* = 0.674).

### Image Analysis for Non-measurable Surgical Cavity Enhancement

The patterns and ADC values of the surgical cavity wall enhancement newly detected on post-CCRT MR imaging are summarized in Table 2.

**Table 2:** Imaging characteristics of non-measurable surgical cavity wall enhancement.

	Total (n = 33)	Progression group (n = 18)	Non-progression group (n = 15)	P value
**Surgical cavity wall enhancement pattern**				***0*.*0032***^***a***^
** Thin wall**	16 (48.5%)	4 (22.2%)	12 (80.0%)	***0*.*0016***^***b***^
** Thick wall**	10(30.3%)	9 (50.0%)	1 (6.7%)	
** Nodular wall**	7(21.2%)	5 (27.8%)	2 (13.3%)	
**nADC**				0.219
** mean**	1.288±0.195	1.326±0.208	1.241±0.172	

^a^ The chi-square test showed a statistically significant difference in surgical cavity wall enhancement patterns between the progression and non-progression groups.

^b^ Thin wall enhancement was more commonly observed in the non-progression group than in the progression group.

Among the 33 patients, thin, thick and nodular wall enhancement were observed in 16 patients (16/33, 48.5%), 10 patients (10/33, 30.3%), and 7 patients (7/33, 21.2%), respectively, on post-CCRT MR imaging. Interreader agreement regarding enhancement pattern categorization was substantial (к = 0.731). Furthermore, interreader agreement was excellent (к = 0.833), when we categorized the enhancement patterns into two groups: thin vs. thick/nodular enhancement. Mean interval from gross total resection to the appearance of non-measurable enhancement was 3.6 ± 0.7 months (mean ± SD). A statistically significant difference was shown in surgical cavity wall enhancement patterns between the progression and non-progression groups (*P = 0*.*0032)*. In the progression group, thick (9 of 18 patients, 50.0%) or nodular (5 or 18 patients, 27.8%) enhancement patterns were more frequently observed than thin wall enhancement (4 of 18 patients, 22.2%). In contrast, thin wall enhancement (12 of 15 patients, 80%) was more common than thick (1 of 15 patients, 6.7%) or nodular wall enhancement (2 of 15 patients, 13.3%) in the non-progression group. Thin wall enhancement pattern was more commonly observed in the non-progression group than in the progression group, whereas thick or nodular wall enhancement was more prominent in the progression group than in the non-progression group (*P = 0*.*0016*). Representative patients with thin, thick or nodular enhancement at the surgical cavity wall on post-CCRT MR imaging are shown in Figs 2–4.

Mean nADC value was higher in progression group. However, we could not find significant differences in nADC values between the progression and non-progression groups (1.326 ± 0.208 vs. 1.241 ± 0.172, *P = 0*.*219*). Additionally, on SWI, we observed dark signal intensity at the surgical cavity wall with thin, thick and nodular enhancement in all of the cases, which was suggestive of post-operative hemorrhage.

### PFS according to the Non-measurable Enhancement Surgical Cavity Enhancement Pattern

**Fig 5:**
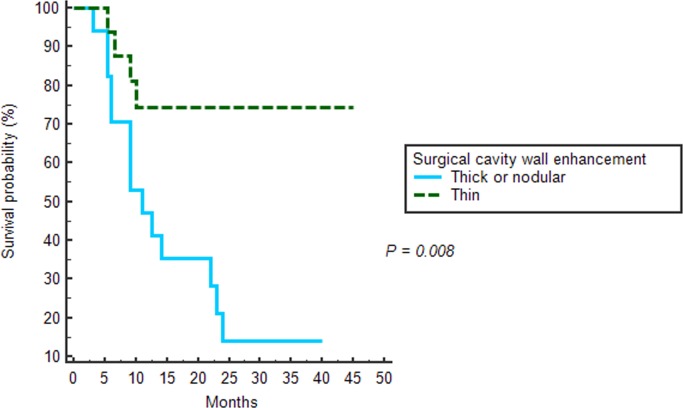
Kaplan-Meier estimates of the progression-free survival according to the surgical wall enhancement patterns of post-CCRT MR imaging.

The average time between detection of new enhancement and determination of progression was 10.2 ± 6.9 months. The mean PFS (Fig 5) of thin wall enhancement group was significantly longer than the nodular or thick wall enhancement group (*P = 0*.*008* by the log-rank test): *35*.*5 months* [95% CI, 27.45–43.56] vs *15*.*8 months* [95% CI, 10.17–21.52]. The median PFS of 17 patients with nodular or thick wall enhancement was 11.0 (6.0–23.0) months.

Among the 17 patients with nodular or thick wall enhancement, 14 patients showed disease progression during the whole follow-up period of the study. Most (13 of the 14 patients) patients showed progression at the site of the previously detected thick or nodular enhancement, except one patient who showed infiltrative tumor progression around the surgical cavity. Nine patients of the 14 patients progressed within one year of follow-up. Particularly, five of the nine patients who progressed within one year developed measurable enhancing lesions during the period of six cycles of adjuvant TMZ. However, only two patients among the four progression group patients with thin wall enhancement developed measurable enhancing lesions during the period of six cycles of adjuvant TMZ. The other two patients with thin wall enhancement progressed within one year of follow-up.

## Discussion

Our study suggested that enhancement patterns of the surgical cavity wall, newly appearing after the completion of CCRT with TMZ following gross total resection, could constitute a significant prognostic factor in GBM patients. One of the most important results from our study was the higher progression rate in patients with thick or nodular wall enhancement pattern than in those with thin wall enhancement patterns (77.8% vs. 20%). Moreover, the mean PFS in the patients with thin wall enhancement pattern was longer than in those with thick or nodular wall enhancement pattern (35.5 vs. 15.7 months). Nine of 17 patients (52.9%) with thick or nodular wall enhancement showed tumor progression over one year of follow-up; in particular, 5 patients showed disease progression during the periods of adjuvant TMZ.

Previous studies examining patterns of recurrence in GBM have shown that 80–90% of tumor recurrences occurred within the radiation treatment field or close to the surgical cavity.[7, 8, 15–18]. Microscopic tumor infiltrations that were not identified by immediate post-operative MR imaging could exist adjacent to the surgical cavity[19, 20]. We hypothesized that tumor recurrence at the surgical cavity might arise from proliferation of microscopically infiltrative tumor cells. Those occult tumor cells can be detected during follow-up period on post-treatment MR imaging, although those were not visible on immediate post-operative images. Thus, our investigation was aimed at determining early MR imaging features that were suggestive of poor prognosis in GBM patients who received standard treatments, which could predict the destination of the microenvironments around the surgical cavity not objectively mentioned by the RANO criteria.

Previous studies have evaluated the patterns of contrast enhancement on early post-operative MR (EPMR) examinations within three days of surgery, following the recommendation of Ekinci et al.[11, 12, 21]. Majos et al.[12] demonstrated that GBM patients with thin-linear enhancement pattern survived longer than those with thick-linear or nodular enhancement patterns (median overall survival: thin linear = 609 days, thick linear = 432 days, and nodular = 318 days). Accordingly, this study suggested that a thin-linear enhancement pattern on EPMR could be considered a benign and favorable finding. Farace et al.[11] showed that more frequent disease progression was observed in patients with partial resection (manifested as residual enhancing lesions on EPMR) than in those who had radical resection (manifested as thin or thick linear enhancement). Our results also supported a good prognosis with the thin linear enhancement pattern at the surgical cavity wall, even on post-CCRT MR imaging in patients who received standard treatment. Other studies [22] have demonstrated ≥5% volumetric increase of the surgical cavity and the surrounding enhancement on post chemotherapy MR imaging in GBM was associated with poor prognosis, suggesting that a small percentage of increase in radiological abnormality may predict prognosis. In this manner, our study demonstrated that newly appearing non-measurable enhancement pattern, which was minimal radiological change, could predict prognosis of GBM patients. Despite the fact that higher progression rate in patients with thick or nodular wall enhancement pattern than in those with thin wall enhancement patterns (77.8% vs. 20%), thick or nodular wall enhancement was not meant to be directly disease progression. Therefore, only the non-measurable enhancement pattern analysis would not have altered treatment plan and excluded patients from the treatment. Nevertheless, early prediction of progression and prognosis of glioblastoma using enhancement pattern may still have some impact on patient treatment, because categorization of non-measurable enhancement pattern is powerful tool for stratifying prognostic groups of patients after completion of CCRT with TMZ. Moreover, radiologists and clinicians can give closer surveillance to patients who have non-measurable thick or nodular enhancement pattern on the post-CCRT MR imaging for early and timely detection of disease progression. Thus, further studies with large cohort on the prognostic impact of the presence of non-measurable enhancement on post-CCRT MR imaging are warranted.

We evaluated the ADC values of enhancing areas at the surgical cavity wall. No significant differences were found between the two groups. In addition, we found dark signal intensity at all of the enhancing portions of the surgical cavity on SWI. Immediate post-operative image findings were assessed to exclude patients with immediate post-operative enhancement. Most of the immediate post-operative images showed post-operative hemorrhage at the resection margin. Although the hemorrhage was barely visible or disappeared over time on post-CCRT T1WI, a rim of dark signal intensity was visualized on SWI, which was suggestive of hemorrhage[23, 24]. Residual blood products, such as hemosiderin, resulted in magnetic susceptibility effects and visualized on SWI could cause a decrease in the value of the ADCs in both the progression and non-progression groups. [25, 26]

Our study had several limitations to be mentioned in addition to the limits of retrospective studies. First, the study population was relatively small. Although we detected statistical significance in the pattern of wall enhancement, further investigations with larger populations are needed to strengthen the statistical power. Second, two different MRI scanners with different field strengths (1.5 and 3.0 T) from different manufactures were used in our study owing to a retrospective study design and the scan parameters were slightly different for each machine. The thickness of wall enhancement could be slightly overestimated in the MR imaging acquired on 3.0 T MR scanner. However, we believe that the effects of the different magnetic field strengths did not significantly affect the classification of wall enhancement pattern from thin to thick/nodular pattern, because we tried to optimize the sequences to maintain the image quality in our institute. The T1WI and contrast enhanced T1WI equally acquired from 1.5 T and 3.0 T scanners, using three dimensional gradient echo sequences with 1mm section thickness without a gap. Third, ROI size used for evaluate ADC value was small (average, 6.91± 6.2 mm^2^). This might lead to the biased calculation, with respect to the limited spatial resolution of ADC maps. Even though manual drawing of the ROI on the enhancing portion is difficult, we drew three ROI onto different sites of the enhancing portion and used average value of ADC to reduce bias. Fourth, in similar terms to the second limitation, we did not include perfusion imaging for the evaluation of the surgical cavity wall enhancements. We believe that non-measurable surgical cavity wall enhancement is too small to be measured on perfusion imaging because of the relatively large voxel size, especially with dynamic susceptibility contrast imaging and arterial spin labeling, so we did not include these modalities.

In conclusion, our results suggested that the pattern analysis of non-measurable surgical cavity wall enhancement on post-CCRT MR imaging might be useful tools for predicting prognosis of GBM patient before clear progression. New, non-measurable, thick or nodular wall enhancement of the surgical cavity wall on post-CCRT MR imaging could be considered an early predictor of disease progression, although MR imaging did not show measurable enhancing lesions.
